# Supplementary material for: Knowledge and attitudes of deaf persons towards safe abortion services in Ghana
Source: PLoS One. 2023 Apr 18;18(4):e0281995. doi: 10.1371/journal.pone.0281995 (PMC10112808; doi:10.1371/journal.pone.0281995)
Supplement: S1 Appendix — (DOCX) [file pone.0281995.s001.docx]

Appendix

Disaggregation of study participants

| **District A** | **District D** |
| --- | --- |
| Female, Focus Group Participant 1, District A  Female, Focus Group Participant 2, District A  Female, Focus Group Participant 3, District A  Female, Focus Group Participant 4, District A  Female, Focus Group Participant 5, District A  Female, Focus Group Participant 6, District A  Female, Focus Group Participant 7, District A  Male, Interview Participant 1, District A  Male, Interview Participant 2, District A  Male, Interview Participant 3, District A | Female, Focus Group Participant 1, District D  Female, Focus Group Participant 2, District D  Female, Focus Group Participant 3, District D  Female, Focus Group Participant 4, District D  Female, Focus Group Participant 5, District D  Female, Focus Group Participant 6, District D  Female, Focus Group Participant 7, District D  Male, Interview Participant 1, District D  Male, Interview Participant 2, District D  Male, Interview Participant 3, District D |
| **District B** | **District E** |
| Male, Focus Group Participant 1, District B  Female, Focus Group Participant 2, District B  Female, Focus Group Participant 3, District B  Female, Focus Group Participant 4, District B  Female, Focus Group Participant 5, District B  Male, Focus Group Participant 6, District B  Female, Focus Group Participant 7, District B  Female, Interview Participant 1, District B  Female, Interview Participant 2, District B  Male, Interview Participant 3, District B | Female, Focus Group Participant 1, District E  Female, Focus Group Participant 2, District E  Female, Focus Group Participant 3, District E  Female, Focus Group Participant 4, District E  Female, Focus Group Participant 5, District E  Female, Focus Group Participant 6, District E  Male, Focus Group Participant 7, District E  Male, Interview Participant 1, District E  Female, Interview Participant 2, District E  Male, Interview Participant 3, District E |
| **District C** | **District F** |
| Female, Focus Group Participant 1, District C  Male, Focus Group Participant 2, District C  Female, Focus Group Participant 3, District C  Female, Focus Group Participant 4, District C  Male, Focus Group Participant 5, District C  Female, Focus Group Participant 6, District C  Female, Focus Group Participant 7, District C  Female, Interview Participant 1, District C  Male, Interview Participant 2, District C  Female, Interview Participant 3, District C | Female, Focus Group Participant 1, District F  Male, Focus Group Participant 2, District F  Female, Focus Group Participant 3, District F  Female, Focus Group Participant 4, District F  Female, Focus Group Participant 5, District F  Female, Focus Group Participant 6, District F  Female, Focus Group Participant 7, District F  Female, Interview Participant 1, District F  Male, Interview Participant 2, District F  Male, Interview Participant 3, District F |
