# Supplementary material for: Knowledge and attitudes of deaf persons towards safe abortion services in Ghana
Source: PLoS One. 2023 Apr 18;18(4):e0281995. doi: 10.1371/journal.pone.0281995 (PMC10112808; doi:10.1371/journal.pone.0281995)
Supplement: Attachment — Submitted filename: Review comments.docx [file pone.0281995.s002.docx]

**General comments**

The paper titled “Usage of safe abortion services among deaf women and girls in Ghana” aims to provide an important contribution to the existing literature on abortion in Ghana, particularly on reproductive health care and the rights of vulnerable women. The paper aimed at enhancing understanding of the experiences of deaf women and girls when accessing safe abortion services. To achieve this, semi-structured interviews and focus group discussions were used and guided by the components of the *accessibility to health theory* by Penchansky and Thomas to collect data for analysis. Findings from the study show gaps in the provision of safe abortion services, especially for vulnerable groups such as women with deafness.

However, there are ethical and several methodological concerns that require explanation and revisions to enhance the quality of the findings. The study used videotaped interviews with permission from the participants. There are usually ethical concerns about videotaped interviews, especially in this case, where audiotape is sufficient. What informed the use of video and not audio? This is troubling, especially on privacy and confidential grounds. The sample used is a vulnerable group and probably coerced for this videotape. Was the videotaping procedure indicated in the protocol approved by Ghana Health Service (GHS)? I also noticed that the ethical approval number from the GHS ethics review committee has not been stated. Kindly incorporate this in the paper.

It is also unclear to me why males were part of the sample, and yet, the title is specifically for women and girls with deafness. The voices of men usually dominate in focus group discussions of both males and females. With this, it could be possible that the findings reflect the opinion of men and not necessarily of women and girls. The sample comprises males and females who did not necessarily induce abortion in their lifetime. The strength of this study rests on experiences and it is unclear how these findings can be trusted, especially when the respondents may not have experienced abortion before. Is not possible that the interviews and focus group discussions reflect what they have heard and necessarily what was experienced? I have provided other comments for consideration based on the sections of the paper.

**Abstract**

1. The statement “The need for policymakers to expedite public education on acceptance of deaf persons as equal members so society” in the conclusion sub-section requires rephrasing. I do not think people with disability are not considered equal members of society. The problem has to do with the designing and programming of interventions. Most interventions often fail to consider the special need of such groups and eventually these people are lopped out of the benefits associated with the interventions.

**Introduction**

2. Revise this statement “Induced abortion, which can be safe or unsafe, is a procedure undertaken intentionally to terminate an unintended pregnancy before the fetus is capable of extrauterine life (1)”. WHO has further categorized unsafe abortion as less safe and least safe. Read the article below and revise.

Ganatra B, Gerdts C, Rossier C, Johnson BR, Tuncalp O, et al. Global, regional, and subregional classification of abortions by safety, 2010–14: estimates from a Bayesian hierarchical model. The Lancet.

2017;390(10110):2372–81.

3. This statement “In fact, there is no study on deaf woman and girls regarding safe abortion practices” requires rephrasing. I do not think the authors have explored studies that are not published or probably in universities' repositories.

**Study participants**

4. How were the districts and regions selected? Kindly be explicit on this. Were the districts/regions selected randomly or purposively?
5. **“**In all, 60 participants took part in this study – six focus group discussions (n=42) and 18 face-to-face interviews were conducted. (See Table 1 below for details)”. This statement seems like results. Kindly move Table 1 to the Results section.
6. Intervals for the age categories in Table 1 are not the same. Revise for the interval to be the same for the first three categories.
7. “The data collection method was deemed appropriate we attempt to develop insight into the experiences of deaf women in their effort to access safe abortion services”. Not clear. Kindly rephrase this statement.
8. “Since not much has been done in this area, the qualitative method was identified as suitable for this study”. This statement contradicts your earlier statement in the introduction section, where you indicated that nothing has been done in this area.

**Procedure**

9. “The study and its protocols were approved by the Ghana Health Services before implementation”. Indicate the approval number from Ghana Health Service Ethics Review Committee.
10. “videotaped”. Refer to my comment on the videotaping.
11. “The interviews and focus groups were conducted between June 2017 and September 2020”. Over three years period for interviewing respondents raises the issue of timing. Three years is a lot of time for significant changes in the demographic, social, economic and reproductive attributes of women. For instance, some of the women within the age bracket "15-25" may not be in the same bracket at the end of the 3 years period.

**Discussion**

12. “we aimed to understand the extent of use of safe abortion services among deaf women and girls in Ghana”. Have the respondents ever used abortion services? So far, it looks like a no answer. In that case, how can we understand the extent of use from a sample that has not experienced abortion or induced abortion?

**Additional comment**

The paper requires proofreading from an English expert. I noticed several grammatical errors that require to be revised to enhance the flow of reading.
