# Supplementary material for: Knowledge and attitudes of deaf persons towards safe abortion services in Ghana
Source: PLoS One. 2023 Apr 18;18(4):e0281995. doi: 10.1371/journal.pone.0281995 (PMC10112808; doi:10.1371/journal.pone.0281995)
Supplement: Attachment — Submitted filename: Response_to_comments.docx [file pone.0281995.s003.docx]

Dear Editor,

Thanks for considering our paper for publication. As you can see, the paper has been edited thoroughly and we are confident all the grammatical errors have been correct. The changes made have been highlighted in colour. Below is our point-by-point response to the review comments.

| Comment | Response | Page number |
| --- | --- | --- |
| Editor |  |  |
| Please ensure that your manuscript meets PLOS ONE's style requirements, including those for file naming. | Thanks for this. We have formatted the paper following the journal’s guidelines. |  |
| You indicated that you had ethical approval for your study. In your Methods section, please ensure you have also stated whether you obtained consent from parents or guardians of the minors included in the study or whether the research ethics committee or IRB specifically waived the need for their consent. | Thanks for this. The ethical approval number has been added. In Ghana, children above 16 years are considered adult and could give consent. However, in this study, those who took part in the study were at least 18 years. We put the 16 years to maintain a consistent range. | See page 13 |
| Please include your full ethics statement in the ‘Methods’ section of your manuscript file. In your statement, please include the full name of the IRB or ethics committee who approved or waived your study, as well as whether or not you obtained informed written or verbal consent. If consent was waived for your study, please include this information in your statement as well. | The ethical approval number has been added. There was three level ethics approval for this study. As you can see, there was institutional approval from a university and two other institutions, GHS and GNAD. The processes have been clarified. | See page 10 |
| Reviewer | Thanks for reviewing our paper and we can confirm including all your suggestions in the revised draft. |  |
| The paper titled “Usage of safe abortion services among deaf women and girls in Ghana” aims to provide an important contribution to the existing literature on abortion in Ghana, particularly on reproductive health care and the rights of vulnerable women. The paper aimed at enhancing understanding of the experiences of deaf women and girls when accessing safe abortion services. To achieve this, semi-structured interviews and focus group discussions were used and guided by the components of the accessibility to health theory by Penchansky and Thomas to collect data for analysis. Findings from the study show gaps in the provision of safe abortion services, especially for vulnerable groups such as women with deafness. |  |  |
| However, there are ethical and several methodological concerns that require explanation and revisions to enhance the quality of the findings. The study used videotaped interviews with permission from the participants. There are usually ethical concerns about videotaped interviews, especially in this case, where audiotape is sufficient. What informed the use of video and not audio? This is troubling, especially on privacy and confidential grounds. The sample used is a vulnerable group and probably coerced for this videotape. Was the videotaping procedure indicated in the protocol approved by Ghana Health Service (GHS)? I also noticed that the ethical approval number from the GHS ethics review committee has not been stated. Kindly incorporate this in the paper. | Thanks for this. The procedure for data collection was approved by IRB and two other institutions, GHS and GNAD. We have provided source to justify the use of videotape to collect data from deaf participants. As you can see, deaf persons communicate using sign language which could be captured using videotape, transcribed and analyzed. As you can see, GNAD was consulted and they were given the study protocol to review before implementation. | See pages 9 and 12 |
| It is also unclear to me why males were part of the sample, and yet, the title is specifically for women and girls with deafness. The voices of men usually dominate in focus group discussions of both males and females. With this, it could be possible that the findings reflect the opinion of men and not necessarily of women and girls. The sample comprises males and females who did not necessarily induce abortion in their lifetime. The strength of this study rests on experiences and it is unclear how these findings can be trusted, especially when the respondents may not have experienced abortion before. Is not possible that the interviews and focus group discussions reflect what they have heard and necessarily what was experienced? I have provided other comments for consideration based on the sections of the paper. | We have justified the inclusion of males in this study. As you can see, deaf marries each other and thus, the need for studies to focus on both groups to understand their experiences and knowledge. | See page 7 |
| The statement “The need for policymakers to expedite public education on acceptance of deaf persons as equal members so society” in the conclusion sub-section requires rephrasing. I do not think people with disability are not considered equal members of society. The problem has to do with the designing and programming of interventions. Most interventions often fail to consider the special need of such groups and eventually these people are lopped out of the benefits associated with the interventions. | Thanks for this. The policies failed to include the needs of individuals with disabilities in societies. We have made recommendation to that effect. | See page 2 |
| Revise this statement “Induced abortion, which can be safe or unsafe, is a procedure undertaken intentionally to terminate an unintended pregnancy before the fetus is capable of extrauterine life (1)”. WHO has further categorized unsafe abortion as less safe and least safe. Read the article below and revise. Ganatra B, Gerdts C, Rossier C, Johnson BR, Tuncalp O, et al. Global, regional, and subregional classification of abortions by safety, 2010–14: estimates from a Bayesian hierarchical model. The Lancet.  2017;390(10110):2372–81. | Thanks for this. The source has been reviewed and included in this study. | See page 3 |
| This statement “In fact, there is no study on deaf woman and girls regarding safe abortion practices” requires rephrasing. I do not think the authors have explored studies that are not published or probably in universities' repositories. | Thanks for this. We have removed the phrase from the revised paper. |  |
| How were the districts and regions selected? Kindly be explicit on this. Were the districts/regions selected randomly or purposively? | We have clarified how the study participants were recruited. The participants were randomly selected. | See page 9 |
| In all, 60 participants took part in this study – six focus group discussions (n=42) and 18 face-to-face interviews were conducted. (See Table 1 below for details)”. This statement seems like results. Kindly move Table 1 to the Results section. | Thanks for this. We moved this section to the result section. | See page 12 and 13. |
| Intervals for the age categories in Table 1 are not the same. Revise for the interval to be the same for the first three categories. | The interval range has been revised for consistency. | See page 13 |
| The data collection method was deemed appropriate we attempt to develop insight into the experiences of deaf women in their effort to access safe abortion services”. Not clear. Kindly rephrase this statement | Thanks for this. The sentence has been revised. | See page 10 |
| Since not much has been done in this area, the qualitative method was identified as suitable for this study”. This statement contradicts your earlier statement in the introduction section, where you indicated that nothing has been done in this area. | Thanks for this. This sentence has been revised from the revised submission. | See page 10. |
| “The study and its protocols were approved by the Ghana Health Services before implementation”. Indicate the approval number from Ghana Health Service Ethics Review Committee. | Thanks for this. The IRB and approvals have been clarified. | See page 10. |
| videotaped”. Refer to my comment on the videotaping. | Thanks for this. The procedure for data collection was approved by IRB and two other institutions, GHS and GNAD. We have provided source to justify the use of videotape to collect data from deaf participants. As you can see, deaf persons communicate using sign language which could be captured using videotape, transcribed and analyzed. As you can see, GNAD was consulted and they were given the study protocol to review before implementation. | See pages 9 and 12 |
| The interviews and focus groups were conducted between June 2017 and September 2020”. Over three years period for interviewing respondents raises the issue of timing. Three years is a lot of time for significant changes in the demographic, social, economic and reproductive attributes of women. For instance, some of the women within the age bracket "15-25" may not be in the same bracket at the end of the 3 years period. | Thanks for this. We decided to maintain the range as supplied by the study participants. The actual processes began in 2017 however, most of the data were collected and transcription were done in 2019 and 2020. In view of this, we decided to maintain the age range supplied by the study participants. |  |
| “we aimed to understand the extent of use of safe abortion services among deaf women and girls in Ghana”. Have the respondents ever used abortion services? So far, it looks like a no answer. In that case, how can we understand the extent of use from a sample that has not experienced abortion or induced abortion? | It is a sensitive issue which was covered in this study. We decided to discuss the issue generally without personalizing it. This gives a snapshot of the experiences of participants who took part in this study. |  |
| The paper requires proofreading from an English expert. I noticed several grammatical errors that require to be revised to enhance the flow of reading. | Thanks for this. The paper has been edited thoroughly. |  |
