# Supplementary material for: Knowledge and attitudes of deaf persons towards safe abortion services in Ghana
Source: PLoS One. 2023 Apr 18;18(4):e0281995. doi: 10.1371/journal.pone.0281995 (PMC10112808; doi:10.1371/journal.pone.0281995)
Supplement: Attachment — Submitted filename: second_response_to_comments.docx [file pone.0281995.s004.docx]

Dear Editor,

Thanks for considering our paper for publication. As you can see, we have incorporated feedback from all the reviewers. Also, a new appendix, disaggregating the composition of participants for FGD and interviews has been added. The changes made has been highlighted in color and below is point-by-point response to the reviewers’ comments.

| **Comments** | **Response** | **Page number** |
| --- | --- | --- |
| **Reviewer 2** |  |  |
| Authors’ efforts are really appreciated but the following comments and recommendations are kindly provided for the stronger presentation about the study. | Thanks for taking time to review our paper and appreciate your feedback on our submission. |  |
| Line number are needed to add according to the PLOS ONE reporting guideline | Thanks for this. We have added line numbers according to PLOS one reporting guidelines. |  |
| Introduction needs in detail about legal abortion services in the study area. It is also necessary to mention the incidence of unsafe abortion among deaf women and girls. | Thanks for this. We have indicated the incidence of abortion among women and girls in Ghana. However, there is no information about what pertains among the study population. It was necessary to provide a bigger picture of what pertains in the study context.  Also, we have provided legal provision made for safe abortion in Ghana. | See page 4 Lines 98 – 106  See page 4 Line 107 – 109 |
| Interconnectedness and intermarriage within the deaf community is not strongly Justified for the present study and to link with the Penchansky and Thomas’ accessibility to health care theory. Therefore, more specific justification is suggested to apply such theory. | Thanks for this comment. Under the theoretical framework, we have outlined reasons or justification for using Penchansky and Thomas’ theory. This is due to the complexity around safe abortion services. | See page 8 Lines 199 - 203 |
| Detailed inclusion and exclusion criteria for focus group discussion FGD should to be included | Thanks for this. We have added the inclusion and exclusion criteria. | Se page 10 Lines 247 - 250 |
| It is good to mention why both males and females were selected for the same FGD section. | The main reason was to achieve convergence. As we have already mentioned, the deaf community is very close-knit and healthy relationship between male and females. GNAD gave consent to both groups participating in the FDG. Also, participants consented to participating in the FDG. | See pages 10-11 Lines 255 - 261 |
| It will be better to mention whether menopaused women participated in this study because their interest may be different from that of the younger aged groups. | We did not really pay attention to this. We gave opportunity for all to participate in this study. |  |
| The detailed procedure of FDG should be described in data collection section such as the facilitator role, number of note taker and their role. | Thanks for this. We have indicated in the procedure how the data were collected from the study participants. | See page 12 Lines 287 – 294 |
| The aim of face-to-face interview in addition to FGD is also needed to explore. | The main reason was to achieve convergence. The same instrument was used for data collection and our aim was to assess whether responses will differ. | See page 11 Lines 259 - 261 |
| Are there any specific criteria of participants for face-to-face interview? | The criteria were those who would express interest to participate in the study. We considered the first 10 participants for this phase. While the first seven participated in the FDG, the remaining three were engaged in face-to-face interviews. | See page 10 Lines 253 - 258 |
| Place of data collection should be mentioned because it is important to keep confidentiality and personal information of the participants related to the abortion services. | The data were collected at the GNAD regional office. | See page 12 Line 290 |
| Author is kindly suggested to mention the timing of face-to-face interview like that of FGD. | Both were collected within the same time framework. We moved from district-to-district. | See page 12 Lines 302 – 303 |
| Working definition of tertiary qualification is suggested to include because readers from different areas and countries cannot know. | This has been added at the foot of the table. | See page 15 Line 354 |
| Is there any information about marital status of the participants obtained during data collection and did the investigator consider the effect of either being married or single status on usage of safe abortion services? | Yes, we collected data about the marital status of the study participants. However, we did not consider its effect on responses. | See page 15 Table 1 |
| Quotation about cost of care are described under the title of availability and accessibility. Therefore, these findings should be analyzed and categorized again according to the working definition in the theoretical framework. | Thanks for this. We have looked at the quotations and analysis. However, we believe that that information was correctly placed. Let’s not forget that the components of the framework are interrelated so the possibility for overlaps. In this study, under availability, the participants were talking about knowledge of safe abortion services. In the preceding sections, it was all about lack of knowledge. The few who said they knew about it indicated that finances would not make it possible to request for such services. Finance was being discussed in the context of availability. The same thing applies to the section under accessibility. | See page 17 lines 402 – 405  See page 18 Lines 426 – 433 |
| **Reviewer 3** |  |  |
| This is a very important work. Thank you for addressing all the comments. The article have been written in a very intelligible manner. I wish all the authors good luck with their future work. | Thanks for taking time to review our paper. |  |
| **Reviewer 4** |  |  |
| Thank you for this very interesting paper and important work highlighting the experiences of deaf  women and girls when accessing abortion services. Thank you for addressing the reviewers comments, especially around the ethical implications of videotaping and the reason for its use. The authors have provided a great deal of supporting evidence and socio-cultural context for the study in the introduction, and have used a great theory; Penchansky and Thomas’ accessibility to health care theory, to derive their interview questions and inform their results | Thanks for taking time to review our paper. |  |
| - “With unsafe abortion being a major cause of maternal deaths among women in developing countries, there is a need to explore the extent to which deaf women and girls in Ghana use safe abortion services” I think this statement needs to be rephrased, as it implies that the aim of the study was to explore the prevalence/how much women and girls in Ghana are accessing safe abortion services and their experience with service delivery, whereas it seems the aim is more around the communities perceptions/attitudes towards safe abortion? The revised paragraph on Page 7, section “Utilization of safe abortion services” does seem to clarify this by saying “Therefore, this study sought to gather the views of deaf persons regarding the accessibility of abortion services in Ghana and deaf women’s knowledge of these services”. I would suggest kindly changing the title of the paper and abstract accordingly.  - “Objective: The main aim of this study was to increase understanding of the experiences of deaf women and girls when accessing safe abortion services.” As above | Thanks for your comments which was taken into consideration. We have changed the title to read “Knowledge and Attitudes of Deaf Persons towards Safe Abortion Services in Ghana.” The study objectives have also been revised accordingly. | See page 1 Line 1  See page 2 Lines 42 – 45, Lines 47 – 49; Lines 162 – 163; Lines 194 – 197; Line 226 – 228; |
| An induced abortion is a procedure undertaken intentionally to terminate an unintended pregnancy before the fetus is capable of extrauterine life (1)”. Reference 1 is a better reference for the categories of abortion (safe to unsafe) authors rather than a single definition of abortion. I would suggest that this definition should be updated, as some abortions may occur for an intended pregnancy with changing circumstances, and some late term abortions may occur in some countries in certain circumstances including threat to the pregnant person’s life. An updated definition could be from the CDC such as: “a legal induced abortion is defined as an intervention performed by a licensed clinician (e.g., a physician, nurse-midwife, nurse practitioner, physician assistant) within the limits of state regulations, that is intended to terminate a suspected or known ongoing intrauterine pregnancy and that does not result in a live birth”. | Thanks for this. We have added the CDC’s definition to balance what we already have. | See page 2 Lines 77 - 81 |
| “……..as women in these countries resort to the use of clandestine, risky, unorthodox, and unsafe means to induce abortion, thereby significantly increasing their risk of dying through complications (1-3, 9)”. I would suggest here that some different wording could be used? For example “covert” instead of “clandestine and perhaps a different word for “unorthodox” or even the use of “unorthodox methods”? While the words clandestine and unorthodox do indeed refer to secrecy and alternative respectively, these particular words may infer that the act is “socially unacceptable” as opposed to done in secret due to legal restrictions. | Thanks for this. The revised sentence reads “Consequently, studies have established that most unsafe abortions (used interchangeably with less safe abortions) take place in sub-Saharan African countries, as women in these countries resort to the use of covert, risky, unorthodox methods, and unsafe means to induce abortion, thereby significantly increasing their risk of dying through complications (1-2, 10).” | See page 3 Lines 90 – 94 |
| A reflexivity statement would be great to further explore the researchers positionality and also views and attitudes towards the research topic | We have added our reflexivity. It is useful to add that two of the co-authors are deaf and members of the deaf community and advocates for their members. | See page 14 Lines 327 – 342 |
| Page 10, line 36, We used a qualitative data collection method to gather data that granted insight into the experiences of deaf women in their efforts to access safe abortion services”. This sentence implies that the aim of the study is to interview women who have attempted to access safe abortion services, whereas it seems the aim of the study are more around general accessibility and knowledge. Kindly suggest this sentenced is rephrased, and include the aim of the study as listed above and in the introduction: 1) views of deaf persons regarding the accessibility of abortion services in Ghana 2) deaf women’s knowledge of these services | Thanks for this. The sentence has been revised as follows “In this study, qualitative data method was deemed appropriate to gather data about the views and knowledge of deaf persons regarding safe abortion services.” | See page 11 Lines 261 – 262 |
| Thank-you for updating the document to note the reason for using videotaping of interviews/focus groups. Please provide some information on how and where the videotapes will be stored safely to ensure patient confidentiality. | A OneDrive file was purchase for this project that is where the recordings have been stored. It is only accessible to the research team. | See page 12 – 13 Lines 300 – 303 |
| The interviews and focus groups were conducted between June 2017 and September 2020, with a duration ranging from 40 minutes to 2 hours”. Thank you for addressing the reviewers’ comments re the long duration of data collection. While I acknowledge the process can take years to complete, it may be worth adding this as a limitation of the study in the Discussion – as socio-cultural factors can change over time as noted by the previous reviewer | We have included the duration as a limitation. | See page 26 Lines 651 - 652 |
| - Page 11, “The videotaped data were transferred to a computer for transcription by two research assistants”. Were the research assistants also proficient in sign language? Or did the data collection researchers translate as the focus groups/interviews were conducted? This is an important context to add as it would add an extra level of interpretation of participants views/experiences | Yes, they are proficient in the use of sign language. they were present during the data collection. | See page 12 lines 285 – 292 |
| Thank you for making an amendment to the methods to explain why male participants were included, however, I note that the results feature many quotes from male participants in the study. As noted by a previous reviewer, this should be noted as a limitation in the discussion section, as firstly, due to power imbalances between men and women, there is a chance that women in focus groups did not feel able to speak their views. | Thanks for this. We have added a limitation in this regard. | See page 26 Lines 651 – 657 |
| If there are quotes from female participants that have undertaken the abortion process it would strengthen the results. I would suggest that due to the nature of participants and results, the study introduction and title should be more framed around the deaf community perceptions rather than being about women and girls experiences. | Thanks for this. The title reads ‘Knowledge and Attitudes of Deaf Persons towards Safe Abortion Services in Ghana’ | See page 1 Line 1 |
| Page 22, “Consequently, abortion is strongly abhorred and rejected by many Ghanaians, and this seems to have influenced the perceptions of the study participants” This is an important point, and one that highlights the incongruence between some of the results – for example, if someone does not agree with abortion this would certainly have effected their perception of barriers as the authors have noted. In the results section, such as results under “Accessibility” it may be worthwhile noting the beliefs of participants around abortion at the start, and whether the quotes or findings are in relation to a participant that is pro-safe abortion generally, abortion only in certain circumstances, or that abortion should not be legal. Or if not amending the results section, kindly expand in the discussion on how their perceptions have been influenced and examples of this. | Thanks for this. We chose the second option which was expansion our discussion. Due to culture and religion, safe abortion practices is not touched on. No discussion is made between safe and unsafe abortion practices as all has been placed in the same box. However, there is a sense that participants are open to safe abortion practices which is what is permitted by the laws of Ghana. | See page 24 Lines 594 - 599 |
| Thank you again for the research paper, I look forward to reading a revised manuscript of what could be an excellent addition to the literature. | Thanks for taking time to review our paper. |  |
